# Enhancing Electrochemical Performance of Si@CNT Anode by Integrating SrTiO_3_ Material for High-Capacity Lithium-Ion Batteries

**DOI:** 10.3390/molecules29194750

**Published:** 2024-10-08

**Authors:** Nischal Oli, Diana C. Liza Castillo, Brad R. Weiner, Gerardo Morell, Ram S. Katiyar

**Affiliations:** 1 Department of Physics, University of Puerto Rico-Rio Piedras Campus, San Juan, PR 00925, USA; 2 Department of Chemistry, University of Puerto Rico-Rio Piedras Campus, San Juan, PR 00925, USA

**Keywords:** Si, SrTiO_3_, CNTs, large volume expansion, ion/electron conductivity, lithium-ion batteries

## Abstract

Silicon (Si) has attracted worldwide attention for its ultrahigh theoretical storage capacity (4200 mA h g^−1^), low mass density (2.33 g cm^−3^), low operating potential (0.4 V vs. Li/Li^+^), abundant reserves, environmentally benign nature, and low cost. It is a promising high-energy-density anode material for next-generation lithium-ion batteries (LIBs), offering a replacement for graphite anodes owing to the escalating energy demands in booming automobile and energy storage applications. Unfortunately, the commercialization of silicon anodes is stringently hindered by large volume expansion during lithiation–delithiation, the unstable and detrimental growth of electrode/electrolyte interface layers, sluggish Li-ion diffusion, poor rate performance, and inherently low ion/electron conductivity. These present major safety challenges lead to quick capacity degradation in LIBs. Herein, we present the synergistic effects of nanostructured silicon and SrTiO_3_ (STO) for use as anodes in Li-ion batteries. Si and STO nanoparticles were incorporated into a multiwalled carbon nanotube (CNT) matrix using a planetary ball-milling process. The mechanical stress resulting from the expansion of Si was transferred via the CNT matrix to the STO. We discovered that the introduction of STO can improve the electrochemical performance of Si/CNT nanocomposite anodes. Experimental measurements and electrochemical impedance spectroscopy provide evidence for the enhanced mobility of Li-ions facilitated by STO. Hence, incorporating STO into the Si@CNT anode yields promising results, exhibiting a high initial Coulombic efficiency of approximately 85%, a reversible specific capacity of ~800 mA h g^−1^ after 100 cycles at 100 mA g^−1^, and a high-rate capability of 1400 mA g^−1^ with a capacity of 800 mA h g^−1^. Interestingly, it exhibits a capacity of 350 mAh g^−1^ after 1000 lithiation and delithiation cycles at a high rate of 600 mA hg^−1^. This result unveils and sheds light on the design of a scalable method for manufacturing Si anodes for next-generation LIBs.

## 1. Introduction

Since their commercial debut thirty years ago, lithium-ion batteries (LIBs), a portable, high-density, and Nobel Prize-winning energy storage technology, have become an integral part of modern life and have revolutionized the fields of consumer portable electronics, electric vehicles (EVs), hybrid electric vehicles (HEVs), and large-scale renewable energy storage systems [1,2,3,4]. Despite significant advancements in technology, including an impressive thirty-fold price reduction between 1991 and 2018, the most substantial improvements have predominantly occurred on the lithium-metal-oxide cathode side [5,6,7,8]. The graphite anodes of LIBs, by contrast, have largely remained unchanged. However, the pursuit of higher energy densities and enhanced battery performance has driven extensive research into advanced alternative energy-dense anode materials [9,10,11,12]. The limited specific capacities of commercial graphite anodes (372 mA h g^−1^) [13] and lithium cobalt oxide cathodes (LiCoO_2_) (140 mA h g^−1^) [14,15] are significant barriers to achieving higher performance, driving the exploration of innovative electrode materials. State-of-the-art LIBs utilizing graphite anodes are nearing the upper threshold of their practical specific energy density.

The energy density of LIBs is predominantly influenced by the specific capacity of the electrode materials. Over the past decade, commercial graphite has been the predominant anode material for LIBs due to its low operational potential (~0.1 V vs. Li/Li^+^), cost-effectiveness, and robust cycling stability [16]. However, graphite poses several critical limitations. Primarily, it has a moderate specific capacity, which constrains the overall energy density of LIBs. Additionally, the lithium intercalation potential of graphite (~0.1 V vs. Li/Li^+^) is close to the lithium metal plating potential (~0 V vs. Li/Li^+^), raising significant safety concerns, particularly under conditions of fast charging or low temperatures [16,17,18]. Consequently, the development of alternative anode materials with higher specific capacities and elevated reaction potentials is imperative for advancing LIB technology [19,20,21]. Among these, silicon (Si) anodes have attracted worldwide attention due to their remarkably high theoretical specific capacity, Li_22_Si_5_ (4200 mA h g^−1^) [22] or Li_15_Si_4_ (3579 mAh g^−1^) [23], which is nearly ten times that of conventional graphite (372 mA h g^−1^ vs. Li/Li^+^), their low mass density of 2.33 g cm^−3^ [24], and favorable operating potential (0.4 V versus Li/Li^+^) [25]. When integrated with high capacity and high-voltage cathodes, such as 5 V spinel (LiMn_x_Ni_y_O_4_, LMNO) [26], Ni-rich LiNi_x_Mn_y_Co_z_O_2_ (NMC) [27], or multi-valent conversion cathodes such as metal fluorides [28] and sulfur [29], Si is expected to achieve specific energy levels of 500 W h kg^−1^ or higher at the cell level [30]. Despite these advantages, the practical application of Si anodes poses significant challenges, including substantial volume expansion (>300%) during lithiation–delithiation, the reactivity of the lithium silicide (Li_x_Si) interface, and issues such as delamination and pulverization, which can cause a loss of electrical contact between active materials and current collectors, leading to mechanical degradation and rapid capacity fading [31]. Addressing these challenges is crucial for unlocking the full potential of Si anodes in next-generation LIBs. Hence, to enhance the electrochemical performance of silicon anodes, it is crucial to mitigate the volume expansion effect and boost conductivity.

The full potential of silicon anode materials has yet to be fully realized, indicating a considerable journey ahead. Significant efforts have been dedicated to achieving stable silicon anodes (SiAs), mainly through the design of nanostructures, the synthesis of composite materials, the improvement of polymer binders, and the optimization of electrolytes [32]. Nanostructure design includes zero-dimensional nanoparticles, one-dimensional nanowires (nanotubes), two-dimensional nanofilms, and three-dimensional porous nanostructures [33]. In addition to nanostructure design, significant efforts have been made to optimize electrolyte compositions. Encapsulating the Si interface within nonreactive materials, such as carbon-based substances, polymers, oligomers, and metal oxides, prevents adverse reactions without hindering the lithiation–delithiation processes. Specifically, engineered interfaces, such as ‘yolk-shell’ and ‘pomegranate’ structures, effectively manage the 350% volume expansion during lithiation [33].

Polyvinylidene difluoride (PVDF) binders are prevalent in commercial applications due to their chemical and electrochemical stability [34]. However, PVDF binders present several challenges for Si anodes, including insufficient mechanical stretchability to accommodate significant volume changes of Si particles, weak adhesion to Si particles and the copper current collector, and the necessity of toxic organic solvents such as N-methyl pyrrolidone (NMP) in slurry preparation [35,36]. To address these binder issues, research has focused on developing water-based functional binders that can dissipate the stress on Si anodes. Binders with polar groups, such as carboxymethyl cellulose (CMC), alginate, poly(acrylic acid) (PAA), and poly(vinyl alcohol) (PVA), exhibit strong adhesive forces toward Si particles via hydrogen bonding [37,38,39]. Additionally, combining elastic moieties with polar functional groups in binders imparts the necessary mechanical properties to Si anodes [40,41,42]. Recently, stress-dissipative elastic waterborne polyurethane (PU) binders for Si anodes have shown promise, possessing both polar functional groups and elasticity [43]. However, the challenge of using toxic solvents, such as NMP, remains. Besides, silicon-based anodes for LIBs, despite innovations, such as stress-dissipating conductive polymer binders [44], accordion frameworks for free-standing, high silicon content [45], carbon fiber-confined yolk-shelled structures [46], and structural tailoring via electrospinning technology [47], continue to be more costly and complex to produce.

Efforts to counteract the volume expansion and potential pulverization of Si have also involved the creation of Si-based composite materials. These composites often include conductive additives (e.g., polymer-based hard carbon, graphene, and carbon nanotubes) or inactive materials (e.g., copper and titanium nitride (TiN)) [48,49]. Additionally, novel architectures have been designed to provide buffering space [50]. Despite significant progress, a trade-off persists between electrochemical performance and processing costs, as achieving excellent properties often requires complex and expensive methods, whereas simpler processes yield unsatisfactory performance [51].

In pursuit of low-cost and commercially viable methods, leveraging the large volume expansion of Si has shown potential. In recent times, advanced energy-generating materials, including ferroelectric, pyroelectric, piezoelectric compounds, as well as their composites, have been increasingly employed in the development of cutting-edge energy conversion technologies [52]. Among these, ferroelectric materials have been integrated into Li-ion batteries to enhance electrochemical performance by generating an electric field in response to mechanical stress [52]. For instance, Xue et al. reported a self-charging Li-ion battery using a piezoelectric poly(vinylidene fluoride) (PVDF) film as a separator, where piezoelectricity was induced by external mechanical stress [53]. In contrast, the current study focuses on internal stress-induced piezoelectricity, where the volume expansion of Si is transferred to a piezoelectric material, generating piezoelectric potential [52]. Furthermore, experiments conducted by Byoung-Sun Lee et al. demonstrated that the piezoelectric potential of the ferroelectric material barium titanate (BaTiO_3_) significantly enhances the electrochemical performance of Si nanocomposite anodes by increasing Li-ion mobility, resulting in improved discharge capacity and cycle performance [52]. However, Si@CNT anodes exhibit poor cycling performance [52]. Additionally, Xuanmeng et al. utilized ferroelectric STO in lithium–sulfur batteries and claimed that it can adsorb polysulfides, effectively inhibiting the lithium–sulfur shuttle and reducing volume expansion during cycles through the ferroelectric effect, leading to impressive long-cycle performance [54].

Herein, for the first time, we present the integration of high-purity, commercially available ferroelectric STO with silicon nanopowder and multi-walled carbon nanotubes (CNTs) using a facile solid-state planetary ball-milling technique. The electrochemical performance of the resulting STO@Si@CNT composite was systematically examined for Li^+^ ion intercalation, demonstrating substantial improvements in battery performance. The CNT matrix functions as a mechanical stress mediator, effectively transferring the volumetric expansion strain of Si to STO while preserving the integrity of conductive pathways key for Li^+^ ion transport. STO nanoparticles provide a local ferroelectric potential that is poled by the deformation of nanoparticles during lithiation. It also influences Li^+^ mobility [52,54]. Moreover, the optimization of a water-based sodium carboxymethyl cellulose (CMC) binder, combined with a standard carbonate-based electrolyte (1M LiPF_6_ in EC:DMC, 1:1 wt% + 10% FEC additive), results in an anode exhibiting a remarkably high specific capacity of ~1500 mA h g^−1^, a superior high-rate capability of ~1400 mA h g^−1^, and exceptional cycling stability beyond 1000 cycles. These findings underline STO’s pivotal role in alleviating the volumetric expansion of silicon anodes and enhancing lithium-ion transport, thereby suggesting that it may hold untapped potential for further advancements in silicon anode battery technologies.

## 2. Results and Discussion

**Figure 1:**
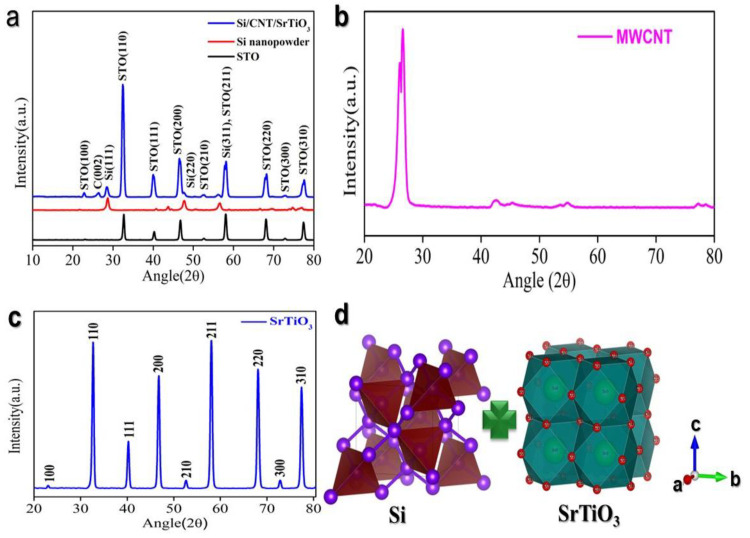
Structural analysis: (**a**) XRD pattern of the Si@STO@CNT composite; (**b**) XRD pattern of CNTs; (**c**) XRD pattern of STO; and (**d**) crystal structure of Si and STO, respectively.

Structural characterization of the Si/CNT/STO nanocomposite was conducted using X-ray diffraction (XRD) analysis. The XRD spectrum, illustrated in Figure 1a, reveals distinct diffraction peaks corresponding to the (100), (110), (111), (200), (210), and (220) crystallographic planes of strontium titanate (STO), as well as the (111) and (220) planes of crystalline silicon. Notably, near 2θ ≈ 58°, the (311) and (211) planes of Si and STO, respectively, nearly overlap. As shown in Figure 1c, the XRD pattern of STO indicates high purity [55]. It is important to highlight that STO can exhibit either cubic or tetragonal crystal structures (Figure 1d), with the cubic phase predominating when the particle size is below 100 nm. Given that the STO particle diameter in this system was below 100 nm, a cubic structure was anticipated. Although distinct diffraction peaks indicative of the carbon nanotube (CNT) microstructure (Figure 1b), such as C (002), were not sharply resolved, the broad feature around 2θ ≈ 26° was ascribed to the presence of CNTs.

**Figure 2:**
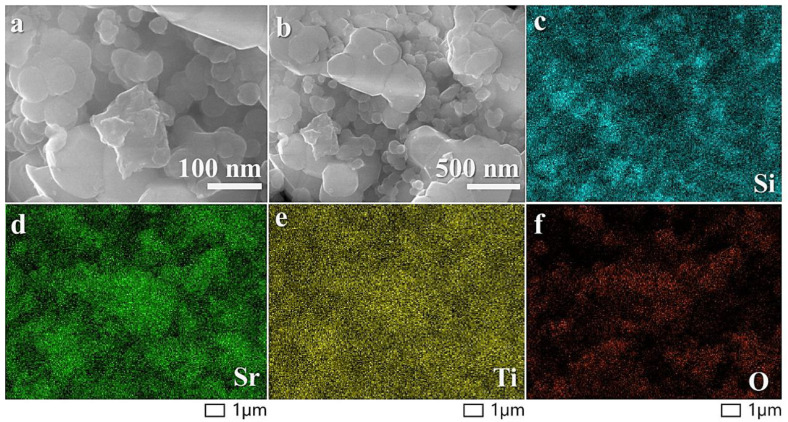
SEM analysis: (**a**) 100 nm; (**b**) 500 nm; and (**c**) SEM mapping of Si; (**d**) Sr; (**e**) Ti; and (**f**) O.

Scanning electron microscopy (SEM) revealed that the nanocomposite surface consists of fragmented CNTs, Si nanoparticles, and STO nanoparticles (Figure 2a,b). The ball-milling process induced CNT breakage, reducing their average length to around 100–150 nm. The SEM image highlights the dense packing of these three components post high-energy ball milling, where the Si and STO regions are distinctly visible and the broken CNTs form a matrix-like framework between them. Furthermore, SEM mapping confirmed a uniform distribution of the Si and STO nanoparticles across the nanocomposite (Figure 2c–f).

**Figure 3:**
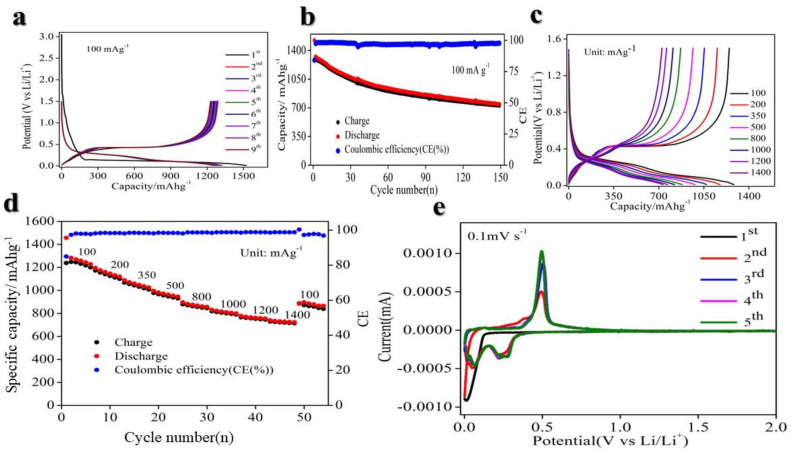
Electrochemical performance of composite Si/STO/CNTs. (**a**) Galvanostatic charge–discharge (GCD) curve at a low current density of 100 mA g^−1^; (**b**) cycling performance at a current density of 100 mA g^−1^; (**c**) rate curve at a range of current densities from 100 to 1400 mA g^−1^; (**d**) rate performance at a range of current densities from 100 to 1400 mA g^−1^; (**e**) cyclic voltammetry at a scan rate of 0.1 mV s^−1^.

The electrochemical performance of the Si/CNT/STO nanocomposite was measured using galvanostatic charge/discharge (GCD) tests. As demonstrated in Figure 3a,e, the voltage curves illustrate stable lithiation and delithiation of Li^+^ ions during thew cycles. Characteristic lithiation and delithiation plateaus of silicon were observed at ~0.25 V during discharge and ~0.5 V during charge. Since STO has a low specific capacity, the overall specific capacity was mainly determined by the Si and CNT content. Despite comprising 30 wt%, CNTs contributed less than 5% to the total specific capacity. Thus, the voltage plateaus were primarily linked to the electrochemical activity of the Si nanoparticles.

The Si/CNTs/STO nanocomposite exhibited behavior similar to Si/CNT composites in the initial cycle, with a reversible capacity of approximately 1510 mA h g^−1^, as shown in Figure 3a,e, and an initial Coulombic efficiency of 84.97%, which stabilized to around 98.3% after subsequent cycling. However, the Si/CNT nanocomposite demonstrated cycling performance degradation upon repeated cycling, attributed to the pulverization of the silicon particles and the subsequent loss of conductive pathways, which has been previously reported [52]. In contrast, the Si/CNT/STO nanocomposites demonstrated enhanced cycling stability, which we attribute to enhanced kinetic behavior resulting from the incorporation of STO, as shown in Figure 3b. As depicted in the voltage profiles in Figure 3e, the addition of STO facilitated the mitigation of performance degradation, supporting more stable lithiation and stable electrochemical performance over time.

In addition to its high capacity and consistent performance, the Si@CNTs@STO anode exhibited remarkable rate capabilities and prolonged cycling stability. As illustrated in Figure 3d, the charge capacities recorded at various current densities of 100, 200, 350, 500, 800, 1000, 1200, and 1400 mA g^−1^ were 1238, 1174, 1068, 978, 880, 818, 766, and 729 mA h g^−1^, respectively. Notably, even at a high current density of 1400 mA g^−1^, the charge capacity remained at 729 mA h g^−1^, which is approximately double the theoretical specific capacity of graphite (372 mA h g^−1^). Upon resuming the original current density of 100 mA g^−1^, the charge capacity recovered to 896 mA h g^−1^, reflecting a recovery ratio of approximately 73%. Figure 3c further depicts the charge/discharge profiles corresponding to these current densities. Additionally, Figure 3b demonstrates the cycling performance at 100 mA g^−1^, where after 150 cycles, the capacity was sustained at 700 mA h g^−1^, with an average Coulombic efficiency of 98.5%.

**Figure 4:**
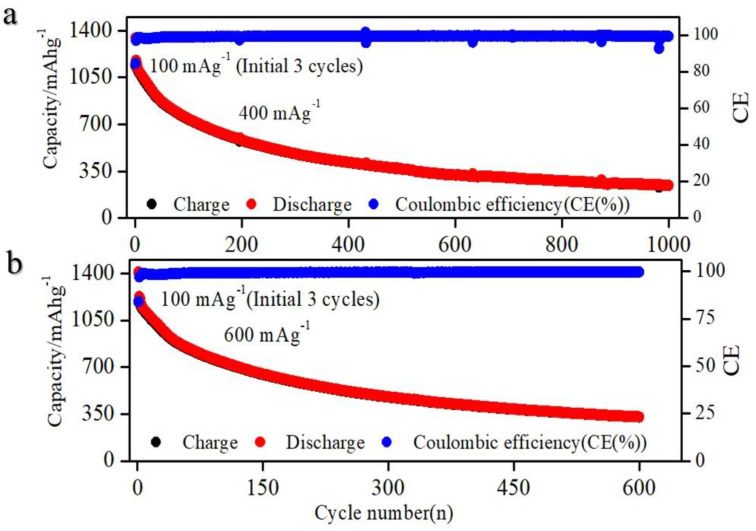
Cyclic performance at current densities of (**a**) 400 mA g^−1^ and (**b**) 600 mA g^−1^.

Figure 4a,b presents the cycling performance at current densities of 400 mA g^−1^ and 600 mA g^−1^, demonstrating consistent stability over more than 1000 cycles. This substantial enhancement in performance can be attributed to the integration of the Si@STO nanocomposite, coupled with the strategic optimization of binders and electrolytes.

Electrochemical Impedance Spectroscopy (EIS) is a sophisticated technique for probing the intricate electrochemical dynamics within LIBs. By measuring impedance, which is essentially the resistance to alternating current across a broad frequency range, a detailed analysis of multiple internal processes is provided. This technique is instrumental in elucidating charge transfer mechanisms at the electrode–electrolyte interface, where Li^+^ ions shuttle between the anode and cathode during the lithiation–delithiation process. It also offers insights into ion diffusion across the electrodes and electrolyte, particularly in the low-frequency region, which is pivotal for understanding overall battery performance. There are distinct key electrochemical parameters contributing to these processes, mainly ohmic resistance (arising from the bulk properties of the electrode and electrolyte materials), charge transfer resistance (Rct) at the electrode–electrolyte interfaces, and double-layer capacitance at the electrode surface. Through careful interpretation of the impedance plot, performance-limiting factors, such as material degradation, internal resistance buildup, and diffusion inefficiencies, are revealed, thereby guiding the optimization of battery materials, architecture, and operational strategies.

**Figure 5:**
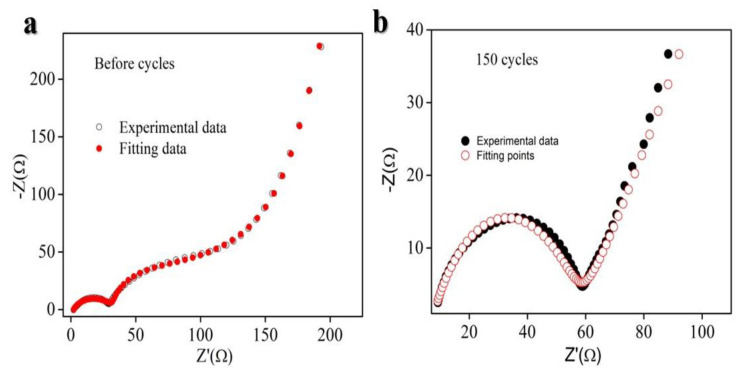
Electrochemical Impedance Spectroscopy analysis of composite Si/STO/CNTs. (**a**) Before lithiation–delithiation and (**b**) after 150 lithiation–delithiation cycles.

As depicted in Figure 5a, the Rct value is lower compared to that observed after 150 cycles in Figure 5b, which con-firms that the incorporation of STO significantly enhances the electrochemical stability and extends the cycling longevity of the system.

## 3. Experimental Setup

### 3.1. Synthesis

The Si@@CNT@STO powder was synthesized utilizing a solid-state route. The nanocomposite maintained a Si/CNT/STO mass ratio of 49:21:30. Precise proportions of commercially sourced precursors, including Si nanopowder, SrTiO_3_, and multiwall carbon nanotubes (Sigma-Aldrich, 99.97% purity, Chicago, IL, USA), were initially ground together using a mortar and pestle for 1 h. Subsequently, these precursors were thoroughly homogenized with a planetary ball mill equipped with zirconia balls (Across International, PQ-N04, Livingston, NJ, USA), operating at 45 Hz (2700 rpm) for 3 h. Finally, the synthesized Si@CNT@STO powder was ground to achieve the desired consistency for further applications.

### 3.2. Structural Analysis

The structural characterization of the synthesized Si@CNT@STO powder was analyzed using an X-ray diffractometer (Rigaku Smart Lab, The Woodlands, TX, USA) with Cu Kα radiation (λ = 1.5408 Å) over a 2θ range of 20° to 80° at a scan rate of 2° per minute. Additionally, morphological assessment and chemical composition analyses were performed using scanning electron microscopy (SEM) and energy dispersive X-ray spectroscopy (EDS) (JEOL, JSM 6480LV, Tokyo, Japan), respectively.

### 3.3. Electrochemical Fabrication and Cell Assembly

In order to perform the electrochemical testing, the anode slurry consisted of a Si/CNT/STO nanocomposite and carboxymethyl cellulose (CMC) binder in distilled water, with a nanocomposite-to-binder mass ratio of 9:1. This slurry was coated on a carbon-coated copper foil substrate (thickness of 9 μm) using a doctor blade (MTI Corporation, Richmond, CA, USA) and was subsequently dried at 80 °C overnight. No additional conductive agent was required as the CNTs within the nanocomposites provided sufficient conductivity. The 1 cm diameter anodes were then obtained from the foil, further dried at 60 °C under vacuum for 3 h, and immediately transferred to the glovebox (MBRAUN, Glovebox Workstations, Stratham, NH, USA). For assembling CR2032 coin-type half-cells, lithium foil was used as the counter electrode and polypropylene ethylene as the separator. For the electrolyte, we utilized 1 M LiPF6 in ethylene carbonate (EC)/diethyl carbonate (DEC) [1:1] with 10% FEC additives. All electrochemical performance tests were carried out using a lithium half-cell in the voltage range of 0.01–1.5 V vs. Li^+^/Li. A cyclic voltammetry (CV) analysis was conducted using various scan rates between 0.1 and 1 mV s^−1^ with an Arbin instrument, College Station, TX, USA. Furthermore, an electrochemical impedance spectroscopy (EIS) analysis was performed using the Arbin instrument. All charge–discharge tests were performed using a Landt battery tester.

## 4. Conclusions

Our investigation revealed that the incorporation of STO enhances the electrochemical performance of Si/CNT nanocomposite anodes. Experimental data and electrochemical impedance spectroscopy confirmed that STO facilitates improved Li^+^ ion mobility. Consequently, the integration of STO into the Si@CNT anode delivers promising electrochemical performance, with a high initial Coulombic efficiency of approximately 85%, a reversible specific capacity of ~800 mA h g^−1^ after 100 cycles at 100 mA g^−1^, and an impressive high-rate capability of 1400 mA g^−1^, maintaining a capacity of 800 mA h g^−1^. Remarkably, the anode retained a capacity of 350 mA h g^−1^ even after 1000 lithiation–delithiation cycles at a high rate of 600 mA g^−1^. These findings highlight a scalable pathway for the design and production of Si-based anodes, offering significant potential for next-generation LIBs.

## Data Availability

The data presented in this study are available on request from the corresponding author.
